# Stream fish metacommunity organisation across a Neotropical ecoregion: The role of environment, anthropogenic impact and dispersal-based processes

**DOI:** 10.1371/journal.pone.0233733

**Published:** 2020-05-26

**Authors:** Pedro Paulino Borges, Murilo Sversut Dias, Fernando Rogério Carvalho, Lilian Casatti, Paulo Santos Pompeu, Mauricio Cetra, Francisco Leonardo Tejerina-Garro, Yzel Rondon Súarez, João Carlos Nabout, Fabrício Barreto Teresa

**Affiliations:** 1 Laboratório de Biogeografia e Ecologia Aquática (Bioecol), Universidade Estadual de Goiás, Anápolis, Goiás, Brazil; 2 Departamento de Ecologia, Universidade de Brasília, Distrito Federal, Brasília, Brazil; 3 Laboratório de Ictiologia, Setor de Zoologia, Instituto de Biociências, Universidade Federal de Mato Grosso do Sul, Campo Grande, Mato Grosso do Sul, Brazil; 4 Departamento de Zoologia e Botânica, UNESP, São José do Rio Preto, São Paulo, Brazil; 5 Laboratório de Ecologia e Conservação de Peixes, Universidade Federal de Lavras, Lavras, Minas Gerais, Brazil; 6 Departamento de Ciências Ambientais (DCA), Universidade Federal de São Carlos, São Paulo, Brazil; 7 Centro de Biologia Aquática, Pontifícia Universidade Católica de Goiás, Goiânia, Goiás, Brazil; 8 Laboratório de Biodiversidade-PPSTMA, UniEVANGÉLICA, Anápolis, Goiás, Brazil; 9 Centro Integrado de Análise e Monitoramento Ambiental (CInAM), Universidade Estadual do Mato Grosso do Sul, Campo Grande, Mato Grosso do Sul, Brazil; Universidade Regional Integrada do Alto Uruguai e das Missoes, BRAZIL

## Abstract

Understanding how assemblages are structured in space and the factors promoting their distributions is one of the main goals in Ecology, however, studies regarding the distribution of organisms at larger scales remain biased towards terrestrial groups. We attempt to understand if the structure of stream fish metacommunities across a Neotropical ecoregion (Upper Paraná—drainage area of 820,000 km^2^) are affected by environmental variables, describing natural environmental gradient, anthropogenic impacts and spatial predictors. For this, we obtained 586 sampling points of fish assemblages in the ecoregion and data on environmental and spatial predictors that potentially affect fish assemblages. We calculated the local beta diversity (Local Contribution to Beta Diversity, LCBD) and alpha diversity from the species list, to be used as response variables in the partial regression models, while the anthropogenic impacts, environmental gradient and spatial factors were used as predictors. We found a high total beta diversity for the ecoregion (0.41) where the greatest values for each site sampled were located at the edges of the ecoregion, while richer communities were found more centrally. All sets of predictors explained the LCBD and alpha diversity, but the most important was dispersal variables, followed by the natural environmental gradient and anthropogenic impact. However, we found an increase in the models’ prediction power through the shared effect. Results suggest that environmental filters (i.e. environmental variables such as climate, hydrology and anthropogenic impact) and dispersal limitation together shape fish assemblages of the Upper Paraná ecoregion, showing the importance of using multiple sets of predictors to understand the processes structuring biodiversity distribution.

## Introduction

Despite advances in understanding the patterns and processes involved in the distribution of aquatic organisms’ at large scale, both in space and time, and the factors responsible for its distribution [1, 2, 3, 4], most studies remain focused on terrestrial groups [5]. Even in the Neotropics, which has a high diversity of freshwater fish [1, 6], there persists a lack of studies on the distribution of aquatic organisms at large spatial scales [7]. Knowing the distribution of aquatic organisms and their structuring factors at large scale, besides providing theoretical advances, also has implications for biodiversity management and conservation [8, 9, 10].

Among the multiple drivers influencing the distribution of aquatic organisms, both dispersal- and niche -based processes are of high importance [11, 12]. Stream and riverine ecosystems are dendritic networks [13] in which the dispersal of strictly aquatic organisms is restricted to the river branches [14]. In these systems, central and more connected sites tend to show similar assemblages via mass effect [13, 15, 16] compared to distant and less connected ones. Overall, headwaters streams located in up-stream portions of a given catchment are less connected sites and therefore tend to receive fewer colonists from the central pool of species and thus show lower species richness [12, 15, 16]. Furthermore, at the basin scale, drainage boundaries may also constrain the dispersal of organisms, resulting in spatial structure of metacommunity [17, 18]. This is even more evident for strictly aquatic organisms, such as fish, as the drainages boundaries may represent barriers to dispersal, resulting in isolation and differentiation of biotas through biogeographic processes (e.g. speciation and extinction) [19, 20]. Therefore, connectivity of sites at the network and organisms’ dispersal capacity are key elements of spatial structure of fish metacommunities [21, 22, 23].

There are, on the other hand, theoretical and empirical evidences indicating an important contribution of environmental (i.e., niche-based) processes in explaining the distribution of stream assemblages [24, 11, 25, 26]. Aquatic organisms are affected by local environmental factors such as habitat variables (e.g. substrate composition, hydrological features, and cover) [27, 28, 29, 30], topographic variables (e.g. latitude, slope) [31, 30] and even current and past climate conditions [1, 32, 33, 34]. Large scale studies have established the importance of climatic variables by demonstrating that these variables can be good predictors of the occurrence of organisms [eg. 35, 1]. Furthermore, it is known that macroscale variables are useful surrogates for local variables [e.g. 36, 37]. Another driver of aquatic biodiversity is human modification of the drainage basin [38, 39]. Several impacts on aquatic organisms, such as deforestation, have been widely reported in the literature as a key driver of species assemblages and one of the main causes of biodiversity loss [40, 41, 28, 42]. This effect is even greater on stream organisms because of their strong and dynamic dependence between aquatic and terrestrial environments [43]. As the landscape degradation intensifies, the unfavourable environmental conditions constrain the occurrence of specialist species, which are progressively replaced by the tolerant/generalist ones [44].

Recent ecological studies have been performed to understand the relative importance of both dispersal- and niche- based processes in determining the distribution of organisms, and some contrasting results have been highlighted. Although niche-based processes are historically recognised as key drivers [45], studies have shown that dispersion of organisms are of primary importance [e.g. 46, 47, 32, 48] whereas others show importance of both processes (niche and dispersal in assemblage organization) [e.g. 49, 29, 3]. However, context dependency appears to be a rule, with the importance of these processes depending on the dispersal capacity of the organisms, the degree of connectivity of sites, spatial extent and environmental heterogeneity which makes it necessary to continue evaluating the factors that determine the distribution of fish assemblages [11, 58, 50, 16, 51].

Therefore, evaluating the relative contribution of multiple processes for distinct organisms and distinct scales is fundamental for understanding how the assemblage is structured and in directing efforts for conservation of aquatic biodiversity, and management measures for places requiring restoration [52, 53, 11, 3]. However, there is currently no study using this approach on a broader scale focusing on stream fish in the Upper Paraná basin, which is one of the Brazilian ecoregions with the highest human population density and highest fish diversity. Therefore, it is vital to understand how fish assemblages are structured, in order to support political decision-making which may affect freshwater habitats within this ecorregion [53, 8, 10]. Additionally, by protecting fish assemblages and freshwater environments, other species are also protected [52], thereby maintaining essential ecosystem services, such as food, climate regulation and fresh water for populations living within the ecoregion [54, 53].

In this study, we seek to understand the processes underlying the stream fish metacommunity organisation in the Upper Paraná ecoregion. Particularly, we evaluate the role of natural environmental gradient, anthropogenic impacts and spatial factors on metacommunity structuring. Given the wide spatial extent, we expect the dispersal processes to be important in explaining the variation in the structure of local communities. Moreover, we expect that secondarily, niche-based processes will also influence metacommunity dynamics so that natural (e.g. climate, topography and hydrology) and anthropogenic (e.g. land use, human population density) gradients will increase the explanatory power of models. We also expect the shared effects between spatial and environmental variables to be substantial, reflecting the spatial structure of environmental conditions across the ecoregion.

We tested these hypotheses by evaluating local fish communities regarding two dimensions: local beta diversity and alpha diversity. The former may be represented by how unique each site is in terms of species composition compared to the set of sites (i.e. LCBD–Local Contribution to Beta Diversity) [55]. Together with alpha diversity descriptors (e.g. species richness), it reveals patterns generated by metacommunity dynamics. For example, assemblages with low species richness and high LCBD (high uniqueness) illustrate an expected pattern in assemblages structured by dispersal limitation. This pattern might be expected in isolated headwaters and those streams located marginally at the basin. Better connected sites may exhibit an opposing pattern, with higher richness despite low uniqueness [18]. Multiple factors may also change species richness and LCBD patterns irrespective the position of sites within the stream network. For example, sites with a high number of species and low uniqueness are expected in degraded regions, with high biotic homogenisation, where species tolerant of a wider distribution predominate [56, 41]. On the other hand, sites with high or low species richness but high uniqueness would indicate biodiversity hotspots [55]. Therefore studies using LCBD as a descriptor of local beta diversity together with alpha diversity have increased worldwide and have been applied to groups of organisms (e.g. [57, 58, 2, 59, 60, 61]).

## Materials and methods

### The Upper Paraná ecoregion

**Fig 1:**
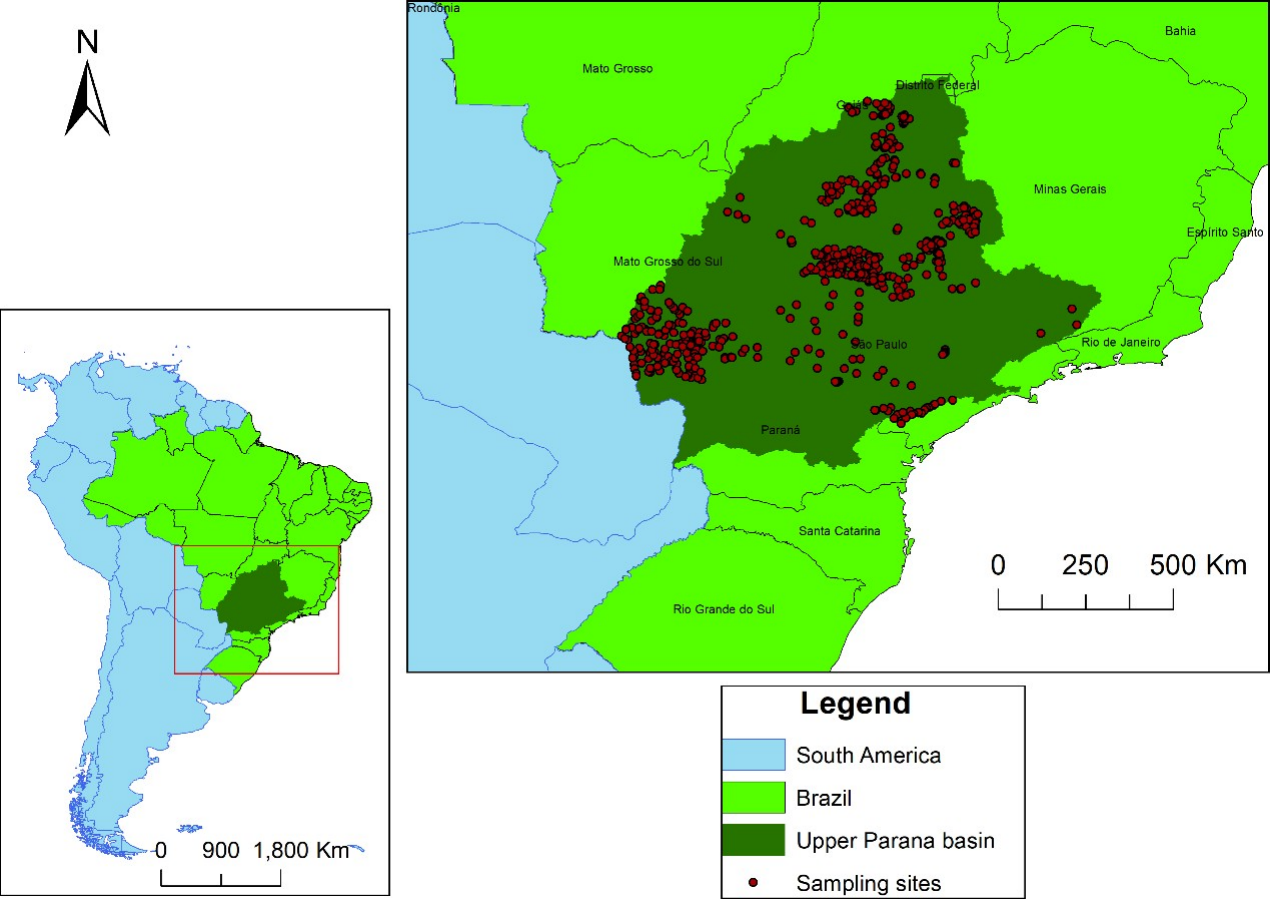
Map of study area showing the Upper Paraná ecoregion in Brazil within South America (left) and sampling sites in the ecoregion (right).

The Upper Paraná ecoregion (UP, ecoregion 344 *sensu* Abell [17]), or upper Paraná River basin, includes the drainages of the upper section of the Paraná River basin, with is the largest hydrographic region in Brazil, forming the Prata or Platina basin (about 48.7% of the surface area [62]), and comprises parts of the Brazilian States of Goiás, Minas Gerais, São Paulo, Paraná Mato Grosso do Sul and the Federal District (Fig 1). The lower-most limit of this area is the Salto de Sete Quedas, a sequence of large waterfalls in the municipality of Guaíra (Paraná State) representing a biogeographic division. After the construction of the Itaipu dam in 1982, the waterfalls were inundated [63] and the reservoir flooded up to 150 km downstream from Salto de Sete Quedas [64]. Today, the artificial division between upper Paraná River and lower Paraná River basins is the Itaipu dam. The upper Paraná River basin is located in one of the most populous and populated regions of Latin America, and consequently it is under intense pressure and demand for natural resources. Despite this, it is a region recognised for its biodiversity, with approximately 310 species of fish [65].

### Biological database

The data used here corresponds to primary (Fild permit number: 34144 (SISBIO-ICMBio); 10327 (SISBIO-ICMBio); 11435 (SISBIO-ICMBio) and 13458–1 (SISBIO-ICMBio)) and secondary data, most of them originating from samplings of fish assemblages carried out in stream reaches by the authors (95%) and a minor portion from data gathered from the literature (5%). Initially, we obtained 1,136 samples of fish, which had been revised to get a more standardised data set according to the following criteria: i) sites with reliable coordinates, ii) samples conducted in first to third-order streams [66], iii) from a single temporal sampling and containing information on fish composition for the point; iv) collection performed in stretches with at least 50 meters of extension; and v) collections carried out with hand-held dragnets or electrofishing. In this way, we retained a total of 586 samples (Fig 1), 28 of the literature and 558 of the authors’ collections.

The fish samplings were performed in stream reaches from downstream to upstream along stretches of at least 50 meters, according to one of the following methods: electrofishing was utilised along a stream reach by researchers holding a net connected to a generator and using the electric discharge to stun/catch fishes [67, 68] or fishes were captured with hand-nets (seines and sieves) carried out by two people along the stream reaches during approximately two hours [69]. The fish caught were fixed in formalin solution and then transferred to 70% ethanol. The final species list obtained from the ecoregion was evaluated by taxonomists specialising in stream fishes (Francisco Langeani and Fernando R. Carvalho) for confirmation of the current species identity, including undefined (i.e., species assigned as sp., spp., aff., cf., and gr.) or doubtful identification (without known distribution in a certain region or in the UP basin).

### Environmental, anthropogenic and spatial data

**Fig 2:**
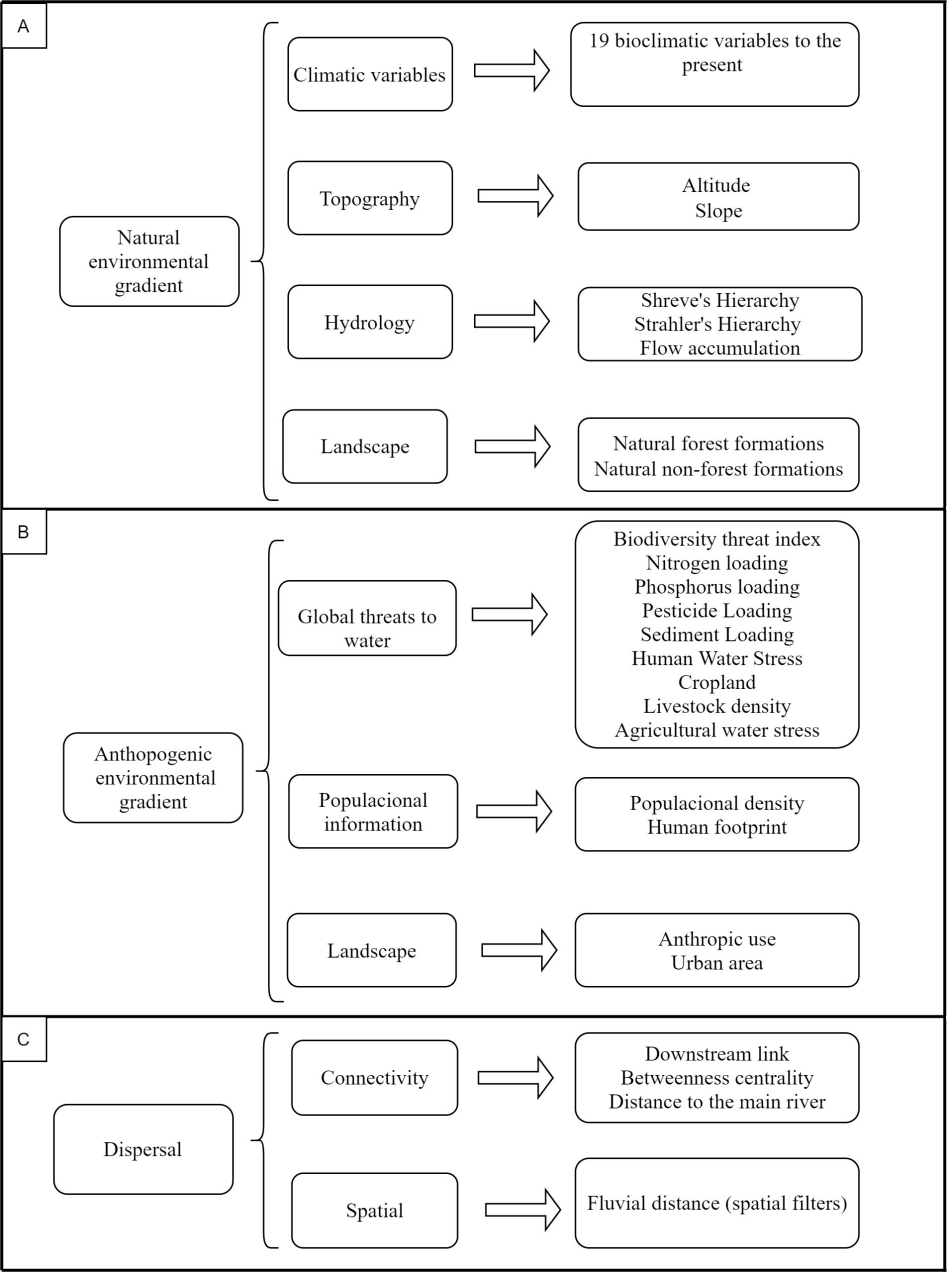
Predictors set used with their respective environmental and spatial variables. A represents the natural environment gradient set; B represents anthropogenic environmental gradient set and C represents dispersal set.

Predictors have been grouped into three sets of variables representing the gradient of environmental, anthropogenic conditions and dispersal (Fig 2). The natural environmental gradient is composed of climatic, topographic and hydrological variables. The climatic variables are composed of 19 bioclimatic variables of current temperature and precipitation obtained from CHELSA (Climatologies at High Resolution for the Earth’s Land Surface Areas) database (http://chelsa-climate.org/), which provides up to date information with grid cells of 1km resolution layers [70]. The topographic variables are composed of altitude and slope (Fig 2A), obtained for cells of 1km of resolution from the earthenv database (http://www.earthenv.org/topography; [71]). The percentage of soil cover by native vegetation in 2016 was obtained from the MapBiomas database (http://mapbiomas.org), considering a buffer of 1km for each point. The dense and open forest classes were grouped into natural forest formation, while non-forest natural humid areas and fields were grouped into natural non-forest formation. For hydrological variables, we generated the Strahler’s [66] and Shreve’s [72] hierarchies using a digital elevation model ASTER with 30 meters of spatial resolution (Fig 2A), and flow accumulation was obtained from the hydroSHEDS database (https://hydrosheds.cr.usgs.gov/; [73]).

Variables describing the anthropogenic gradient represent the incidence and intensity of impacts that potentially affect aquatic ecosystems, such as the aquatic biodiversity threat index, as well as their individual variables [74]; human population density; human footprint; percentage of land used for urban infrastructure and other anthropogenic uses (e.g. agriculture and pasture (Fig 2B)). The biodiversity threat index and the variables composing it were obtained from the river threats database (http://www.riverthreat.net/; [74]). Human population density per municipality was obtained from the IBGE (Instituto Brasileiro de Geografia e Estatística) (http://www.ibge.gov.br) website and human footprint from SEDAC [75]—Socioeconomic Data and Applications Center (http://sedac.ciesin.columbia.edu/data/set/wildareas-v2-human-footprint-geographic). The percentage of anthropogenic land use (pasture, annual crops, semi-perennial crops, agriculture or pasture and non-vegetated areas) and urban infrastructure were quantified using the same procedures used to describe the natural forest and non-forest formations described above.

Spatial variables were selected to represent different connectivity processes (e.g., dispersal among assemblages via river course; upstream dispersal through main river). The spatial variables included the downstream link, betweenness centrality, distance to main river (sixth order) and the fluvial distance (PCNM—Principal Coordinates of Neighbour Matrices [76], obtained from the pairwise distance between streams via watercourse (Fig 2C). The downstream link was obtained from Shreve’s hierarchy; the points were moved to the nearest upper confluence, avoiding first-order segments [77]. Betweenness centrality, which is a connectivity/centrality information, is represented by the number of paths that are connected to a particular place of interest, so local assemblages that are more central in the basin receive a higher value because they are more likely to be connected with other assemblages [78]. For the distance to the main river, we used the Strahler’s hierarchy, calculating the distance via the nearest major river course (sixth order) for each sample point. PCNMs were derived from a fluvial distance matrix between the sample points via watercourse [79], since the fluvial distance has a good predictive power to represent the dispersion of aquatic organisms at different geographic scales, where the first PCNM indicated the relationship among sites at broad scale, and the last PCNM at finer scale [80, 81].

### Biological diversity

Alpha diversity was measured as the species number of each assemblage. The beta diversity was obtained through the decomposition of regional variation in species composition among assemblages (total beta), obtaining the relative contribution of local assemblages to total beta diversity, the LCBD (Local Contribution to Beta Diversity) [55]. LCBD describes the uniqueness of each assemblage in relation to the set of assemblages. It was calculated from the presence/absence matrix of species, using the Sorensen dissimilarity coefficient, which is one of most appropriate for diversity studies [55]. This index varies between 0 and 0.5, where 0 indicates totally similar assemblages and 0.5 indicates totally dissimilar assemblages [55].

### Data analysis

We calculated the Variance Inflation Factor (VIF) and removed collinear variables (i.e. VIF values > 10) within each of the three variables sets (natural, anthropogenic and spatial environmental gradient) using the *vifstep* function of the *usdm* package [82]. After selecting the non-collinear variables (see S1 and S2 Tables), each set of variables was standardised (mean = 0, standard deviation = 1). Moreover, to reduce the number of spatial predictors generated (478 variables), we tested a previous selection of the most important variables to predict the patterns of alpha diversity and LCBD using the *forward selection* procedure until the overall p value reached 0.05 as a stopping criteria (see S3 and S4 Tables, *packfor* package [83]). Then, we tested a global regression model for each set of predictors separately and the significant set of predictors (p < 0.05) were used in a variance partitioning analysis [84].

We calculated the variance partitioning to evaluate the relative contribution of anthropogenic, natural and spatial gradient on LCBD and alpha diversity, using the *varpart* function of *vegan* package [85]. The regression components were: A- variation explained only by anthropogenic environmental gradient; B- variation explained only by natural environmental gradient; C- variation explained only by space; D- variation shared between anthropogenic and natural environmental gradient; E- variation shared between anthropogenic environmental gradient and space; F- variation shared by natural environmental gradient and space; G- variation shared by natural, anthropogenic environmental gradient and space; and H- residual variation [86, 87]. Afterwards, we performed a regression for each group to test the significance of fractions A, B and C, controlled by the other groups and the response variables (LCBD and alpha diversity), followed by an Analysis of Variance (ANOVA) for each component. We also performed multiple linear regressions for each set of predictors and each response variable to determine, from among the set of variables, which was most important in order to explain the variation in species richness and LCBD. All data analyses have been performed in R environment [88].

## Results

We registered 177 species; the most representative order was Characiformes (73 species), followed by Siluriformes (68; see S5 Table). The species with the greatest absolute frequency number in the basin was *Hypostomus* cf. *ancistroides* (307 individuals), followed by *Astyanax lacustris* (288), *Rhamdia* aff. *quelen* (247) and *Astyanax fasciatus* (235; see S5 Table for occurrence data for all species).

**Fig 3:**
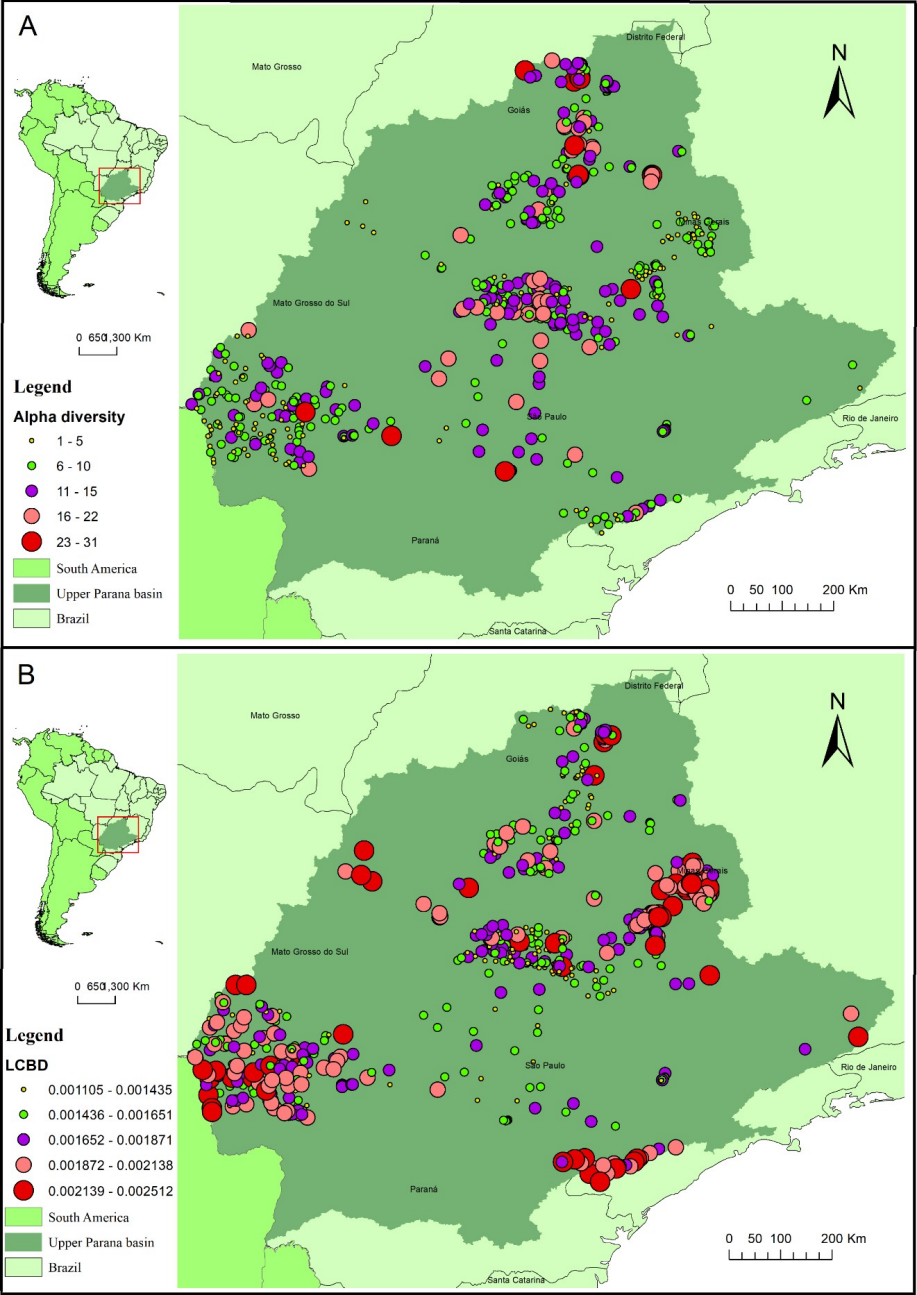
Map with values of alpha diversity (A) and LCBD (B) of sampling sites in the Upper Paraná ecoregion.

The alpha diversity varied from 1 to 30. The beta diversity for the whole basin region was high (0.41), with LCBD from local assemblages varying from 0.0011 to 0.0025. Overall, sites with higher contribution to beta diversity are located in peripheral regions of the basin (Fig 3), and sites with lower alpha diversity were the most important contributors to beta diversity. In fact, we found a negative correlation between LCBD and alpha diversity (Pearson correlation: r = -0.63; p<0.001).

According to the global models, the three sets of predictors were significantly associated with alpha diversity and LCBD (p<0.05). For alpha diversity, the effect solely of space was the most important (35%), followed by the natural environmental gradient (19%) and the anthropogenic environmental gradient (12%). The spatial variables also showed the greatest explanation power of LCBD (43%), followed by natural environmental gradient (26%) and anthropogenic (20%). All predictors explained together 47% of LCBD and 43% of alpha diversity (S6 and S7 Tables).

**Fig 4:**
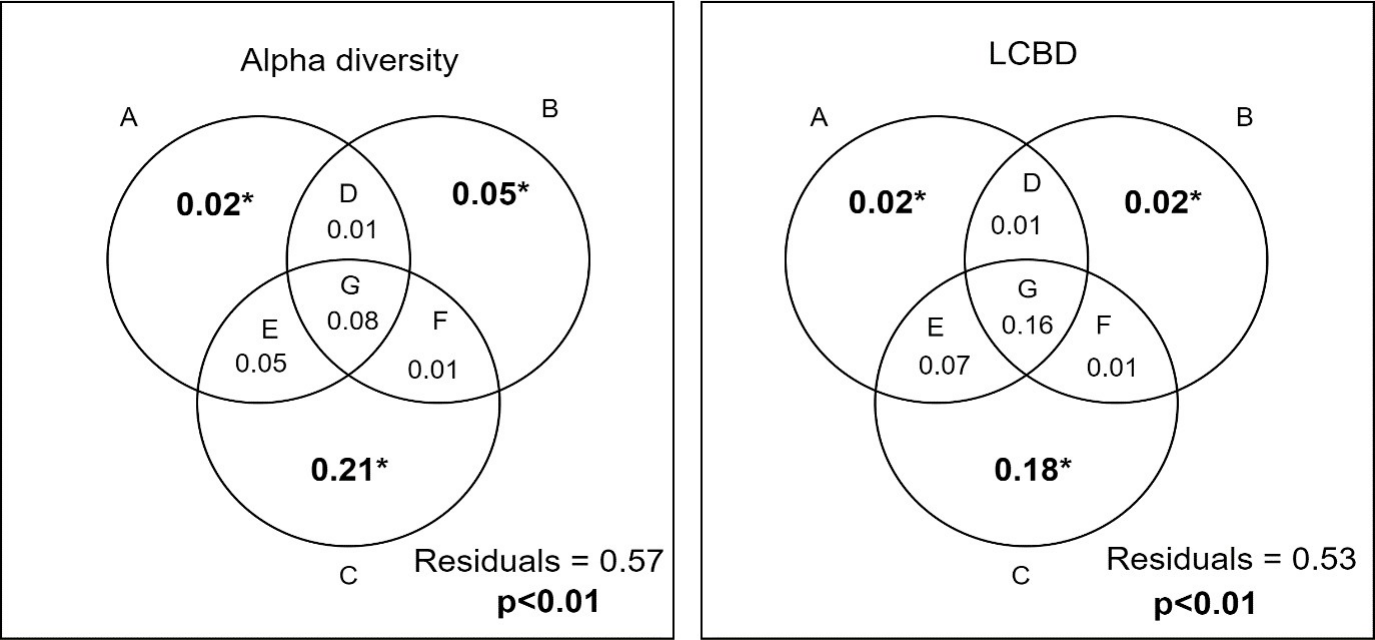
Results of variance partitioning of alpha diversity and LCBD. A- anthropogenic environmental gradient; B- natural environmental gradient; C- Space; D- shared effect of anthropogenic and natural environmental gradient; E- shared effect of anthropogenic environmental gradient and space; F- shared effect of natural environmental gradient and space; G- shared effect of natural, anthropogenic environmental gradient and space. Bold values with * indicate significant set of predictors (p<0.05).

The variance partitioning showed that the explanation patterns of LCBD and alpha diversity regarding the predictors were similar, which were explained mainly by the spatial component (Fig 4), however with different processes, as highlighted by the negative relationship among these two variables. The variances explained by purely environmental factors, although significant, were small for both metrics. We highlighted the interaction between sets of predictors (shared explanations) that added explanation power to the models of LCBD and alpha diversity, mainly the interaction between the three groups of variables (Fig 4 and S6 and S7 Tables).

For the spatial component, the multiple regression demonstrated that LCBD and alpha diversity presented opposing effects, where positive effect of one predictor on LCBD was negative for alpha diversity. For example, the broad PCNM 6, presented positive association with alpha diversity, and negative association with LCBD. Both LCBD and alpha diversity had significant relationships with spatial filters, created by fluvial distance matrix, describing different scales (broad and finer scale).

Three variables of anthropogenic gradient were important for both alpha diversity and LCBD, while six variables of natural environmental gradient were important for alpha diversity and five for LCBD (Table 1). Among the anthropogenic gradient variables, livestock density and urban infrastructure showed a negative relationship with alpha diversity, such as phosphorous transportation and anthropogenic land use, which were negatively associated with LCBD. In other cases, especially in the natural environment data set, we found divergence between the associations of variables with alpha diversity and LCBD (Table 1). The other associations between predictors and response variables are detailed in S8 and S9 Tables.

**Table 1:** Results of multiple regressions highlighting the predictor variables significantly associated with species richness and LCBD in order of importance (for more details of coefficients see S8 and S9 Tables). Non-bold values indicate a positive association, while bold values indicate a negative association with the response variable.

Variables	Alpha diversity	LCBD
Spatial	PCNM 6, 1, *Betweenness centrality*, PCNM 95, 123, 25, 441, 16, 46, 361, 12, 8, 38 and PCNM 2. **PCNM 4, 5, 58, 59, 44, 139, 85, 232, 358, 82, 155, 33, 62, 315, 43, 20, 50, 55, 174 and PCNM 142.**	PCNM 5, 4, 11, 59, 33, 60, 155, 247, 232, 85, 96, 58, 327, 268, 104, 62, 146, 223, 314, 228, 424, 41, 82, 107, 24, 112 and PCNM 50. **PCNM 10, 6, 1, 7, 16, 8, 17, 250, 30, 144, 57, 211, 148, 231, 238 and PCNM 31.**
Anthropogenic	Phosphorus Loading	Sediment Loading.
	**Urban infrastructure and Livestock Density.**	**Phosphorus Loading and Anthropogenic use.**
Natural	Bio 03 (Isothermality), Bio 09 (Mean Temperature of Driest Quarter), Bio14 (Precipitation of Driest Month), Flow accumulation and Strahler's Hierarchy.	Bio 18 (Precipitation of Warmest Quarter) and forest formations.
		**Bio 03 (Isothermality)**, **Bio 09 (Mean Temperature of Driest Quarter) and Bio 14 (Precipitation of Driest Month).**
	**Bio 02 (Mean Diurnal Range).**	

## Discussion

We evaluated the environmental, anthropogenic and spatial drivers of alpha and beta diversity of headwater streams at the regional scale. The Upper Paraná ecoregion is composed of distinct stream fish assemblages, as evidenced by the high total beta diversity values, and high uniqueness on the ecoregion edges, which suggests local endemism. Corroborating our first hypothesis, spatial variables are more important to explain alpha diversity and local beta diversity variation. In addition, natural and anthropogenic environmental gradients added explanatory power through the shared effect, which is in accordance with our second prediction. The results indicate that both dispersal limitation, environmental filters and anthropogenic drivers are important to shape the distribution of fish assemblages in the Upper Paraná ecoregion.

Corroborating our first prediction, the spatial variables explained most of the variation in alpha diversity and LCBD. In general, spatial filters of broad scales (i.e., first PCNMs) were the most important in explaining richness and LCBD variation, which may be associated with the isolation of biotas within the ecoregion [11]. Therefore, biogeographic processes (e.g. speciation, extinctions and headwater capture) could result in the ichthyofauna regionalisation that could be explained, for example, by the isolation of assemblages that drain large rivers (e.g. Tietê, Paranapanema, Peixe, São José dos Dourados, Grande, Sucuriú), all of them connected by the Paraná River. This seems to represent a barrier to low-order stream species migration and leads to the isolation of these sites [89].

Assemblages with greater uniqueness and lower species richness were located at the basin edges. These streams probably experience higher isolation, and colonisation may be restricted, resulting in lower richness and differentiation in assemblage composition [16]. On the other hand, assemblages located centrally would be more easily colonised and would thus exhibit higher similarity. This process may be exemplified by the positive relationship between richness and the degree to which a stream is connected within the hydrological network (measured by betweenness centrality). Despite the importance of ecological processes, the uniqueness of streams located at basin borders could also be interpreted as an evidence of historical processes such as headwater captures. In fact, headwater captures between the Upper Paraná and adjacent basins, such as Upper Tocantins, Upper São Francisco, Coastal drainages and Upper Paraguay were already documented (e.g. [90, 91, 92, 93]). Lima & Ribeiro [20] show that the headwater captures presumably were due to recent tectonic events that the ecoregion has been experiencing over time and which facilitated species exchange between the subjacent basins. Indeed, there is a greater relationship and similarity between neighbouring drainage headwaters fauna of non-affiliate basins than between drainages which share the same basin [94]. Finer-scale spatial processes, represented by mass effects and patch dynamics within the ecoregion sub-basins might also explain the spatial structure of alpha diversity and uniqueness [11]. In fact, some finer-scale spatial filters were also important explanatory variables of richness and LCBD. Therefore, the spatial signal in alpha and beta diversity we found for the Upper Paraná fish assemblages may be explained by historical processes, as well by ecological processes (i.e. dispersal limitation, mass effects and patch dynamics) of the metacommunity theory [95, 96, 97].

According to our second hypothesis/prediction, the explanation attributed to environmental predictors (natural and anthropogenic) added a powerful explanation through the shared effect. This suggests that environmental conditions are spatially structured, which would also have an impact on the differentiation patterns of assemblages through niche processes [98, 99]. In fact, less differentiated assemblages are located in more degraded and central areas (e.g., the state of São Paulo). These streams with a higher degree of anthropisation in the basin would shelter poorer assemblages with less species differentiation, suggesting biotic homogenisation in these regions is mediated via the colonisation of streams by tolerant, generalist and widely distributed species [44] as has been observed in other studies (e.g. [41, 100, 101, 102]).

The negative relationship between LCBD and alpha diversity found here has been recorded in literature, and suggest that richer sites do not necessarily shelter more unique species [55, 103, 2, 59], although this relationship is not always negative [55; e.g. 57, 104]. Headwater streams are located at the extremity of the hydrographic network and thus, are more isolated than reaches of greater river hierarchy [105]. Besides, headwater streams have high heterogeneity in their environmental conditions [106]. The low connectivity associated with the particular conditions mean that these streams may present low species richness, through limitation of colonisation, and a greater uniqueness of species composition (higher LCBD). In fact, low-order streams had lower species richness and higher LCBD, and more connected streams (higher betweenness centrality) had higher alpha diversity.

Finally, despite the great number of predictors that are important for fish that were used herein, a fraction of LCBD variation and alpha diversity remain unexplained. This may suggest that other variables (e.g. finer-scale variables and interaction information) may also be important in assemblage structuring [26]. Although we did not include local scale variables, it is notable that macroscale variables can be good surrogates for local variables [36, 37]. Thus, we emphasise that studies should include other sets of predictors to increase the explanatory power of models and should attempt to explain how assemblages are structured in space and time [107]. We also emphasise the importance of using different sets of predictors to understand the distribution of assemblages, since different variables, when combined, can increase the explanatory power of models, as evidenced by the shared fraction of the three sets of predictors in the present study.

## Conclusions

We demonstrated that the Upper Paraná ecoregion has a high beta diversity and that there is a significant degree of predictability in the distribution of alpha diversity and uniqueness throughout the ecoregion, especially in spatial terms. Streams with high uniqueness are distributed in different sub-basins, with high local beta diversity and low alpha diversity mainly in peripheral regions of the basin. Thus, our results highlight the importance of conserving mainly the headwaters streams at the basin edge and at the same time, supporting the need for restoration actions in the central portions of the ecoregion. The limited species dispersal and environmental factors together shape the upper Paraná fish assemblages, demonstrating the importance of multiple processes in the organisation of metacommunities at large scales in the Neotropical region.

## Supporting information

S1 Table Description of VIF values used to eliminate the collinear variables of the anthropogenic environmental gradient component. (DOCX)

S2 Table Description of the VIF values used to eliminate the collinear variables of the natural environmental gradient component. (DOCX)

S3 Table Spatial variables selected by forward selection procedure for LCBD (p < = 0.05). (DOCX)

S4 Table Spatial variables selected by forward selection procedure for alpha diversity (p < = 0.05). (DOCX)

S5 Table List of species with number of occurrences and orders for the Upper Paraná River basin. (DOCX)

S6 Table Results of variance partition for LCBD. X1- Explanation of anthropogenic environmental gradient, X2- Explanation of natural environmental gradient, X3- Explanation of space component, A anthropogenic environmental gradient, B—Natural environmental gradient, C—Space, D—Shared effect between anthropogenic and natural environmental gradient, E—Shared effect between anthropogenic environmental gradient and space, F—Shared effect between natural environmental gradient and space, G—Shared effect between anthropogenic natural environmental gradient and space, H—Residuals. Bold values indicate sets of predictors that were significant (p <0.05).
(DOCX)

S7 Table Results of variance partition for alpha diversity. X1- Explanation of anthropogenic environmental gradient, X2- Explanation of natural environmental gradient, X3- Explanation of space component A—Anthropogenic environmental gradient, B—Natural environmental gradient, C—Space, D—Shared effect between anthropogenic and natural environmental gradient, E—Shared effect between anthropogenic environmental gradient and space, F—Shared effect between natural environmental gradient and space, G—Shared effect between anthropogenic, natural environmental gradient and space, H—Residuals. Bold values indicate sets of predictors that were significant (p <0.05).
(DOCX)

S8 Table Regression between the variables of each predictor set and the LCBD. Bold values indicate predictors that were significant (p <0.05).
(DOCX)

S9 Table Regression between the variables of each set of predictors and alpha diversity. Bold values indicate predictors that were significant (p <0.05).
(DOCX)

S1 File Geographic coordinates (decimal degrees) of the streams, local contribution to beta diversity (LCBD) and Alpha diversity values. (XLS)

S2 File Variables used in the article. (XLS)
