# Supplementary material for: Stream fish metacommunity organisation across a Neotropical ecoregion: The role of environment, anthropogenic impact and dispersal-based processes
Source: PLoS One. 2020 May 26;15(5):e0233733. doi: 10.1371/journal.pone.0233733 (PMC7250414; doi:10.1371/journal.pone.0233733)
Supplement: S1 Table — (DOCX) [file pone.0233733.s001.docx]

**S1 Table. Description of VIF values used to eliminate the collinear variables of the anthropogenic environmental gradient component.**

| Variables VIF |
| --- |
| Biodiversity threat index 8.915232 |
| Nitrogen loading 1.963434 |
| Phosphorus loading 4.012158 |
| Pesticide loading 3.597829 |
| Sediment loading 5.550662 |
| Human water stress 2.274875 |
| Cropland 3.029649 |
| Livestock density 1.460269 |
| Populacional density 1.437738 |
| Human footprint 1.606973 |
| Anthropic use 1.479396 |
| Urban area 1.617646 |
