# Supplementary material for: Stream fish metacommunity organisation across a Neotropical ecoregion: The role of environment, anthropogenic impact and dispersal-based processes
Source: PLoS One. 2020 May 26;15(5):e0233733. doi: 10.1371/journal.pone.0233733 (PMC7250414; doi:10.1371/journal.pone.0233733)
Supplement: S2 Table — (DOCX) [file pone.0233733.s002.docx]

**S2 Table. Description of the VIF values used to eliminate the collinear variables of the natural environmental gradient component.**

| Variables | VIF |
| --- | --- |
| Mean Diurnal Range | 3.670162 |
| Isothermality | 9.855158 |
| Mean Temperature of Wettest Quarter | 6.204585 |
| Mean Temperature of Driest Quarter | 5.953666 |
| Annual Precipitation | 3.211455 |
| Precipitation of Wettest Month | 6.660829 |
| Precipitation of Driest Month | 7.340449 |
| Precipitation of Warmest Quarter | 2.284558 |
| Flow accumulation | 1.211269 |
| Shreve's Hierarchy | 1.686297 |
| Strahler's Hierarchy | 1.604158 |
| Slope | 2.257832 |
| Natural forest formations | 1.816873 |
| Natural non-forest formations | 1.160046 |
