# Supplementary material for: Stream fish metacommunity organisation across a Neotropical ecoregion: The role of environment, anthropogenic impact and dispersal-based processes
Source: PLoS One. 2020 May 26;15(5):e0233733. doi: 10.1371/journal.pone.0233733 (PMC7250414; doi:10.1371/journal.pone.0233733)
Supplement: S3 Table — (DOCX) [file pone.0233733.s003.docx]

**S3 Table. Spatial variables selected by forward selection procedure for LCBD (p <= 0.05).**

| **Variables** | **Order** | **R2** | **R2Cum** | **AdjR2Cum** | **F** | **pval** |
| --- | --- | --- | --- | --- | --- | --- |
| PCNM 4 | 7 | 0.057754 | 0.057754 | 0.05614311 | 35.85683 | 0.000999 |
| PCNM 5 | 8 | 0.046554 | 0.104308 | 0.10124021 | 30.35357 | 0.000999 |
| PCNM 10 | 13 | 0.04058 | 0.144888 | 0.14048735 | 27.66666 | 0.000999 |
| PCNM 1 | 4 | 0.036617 | 0.181505 | 0.17587938 | 26.03705 | 0.000999 |
| PCNM 6 | 9 | 0.033661 | 0.215165 | 0.20841115 | 24.91834 | 0.000999 |
| PCNM 7 | 10 | 0.017878 | 0.233043 | 0.22510918 | 13.5199 | 0.000999 |
| PCNM 16 | 19 | 0.016363 | 0.249406 | 0.24033168 | 12.62225 | 0.000999 |
| PCNM 11 | 14 | 0.015521 | 0.264927 | 0.25475279 | 12.20409 | 0.000999 |
| PCNM 59 | 62 | 0.011899 | 0.276825 | 0.26554536 | 9.493525 | 0.008991 |
| PCNM 8 | 11 | 0.010102 | 0.286927 | 0.27454749 | 8.159982 | 0.005994 |
| PCNM 247 | 250 | 0.008775 | 0.295702 | 0.28222833 | 7.163748 | 0.014985 |
| PCNM 232 | 235 | 0.008423 | 0.304125 | 0.28957717 | 6.947981 | 0.012987 |
| PCNM 60 | 63 | 0.008363 | 0.312488 | 0.29688988 | 6.969905 | 0.004995 |
| PCNM 155 | 158 | 0.008532 | 0.32102 | 0.30440119 | 7.187441 | 0.008991 |
| PCNM 33 | 36 | 0.008943 | 0.329963 | 0.31236136 | 7.621529 | 0.003996 |
| PCNM 17 | 20 | 0.010285 | 0.340248 | 0.32172914 | 8.886232 | 0.008991 |
| PCNM 58 | 61 | 0.0074 | 0.347649 | 0.32815826 | 6.454558 | 0.010989 |
| PCNM 30 | 33 | 0.007186 | 0.354834 | 0.33438873 | 6.326142 | 0.018981 |
| PCNM 96 | 99 | 0.007212 | 0.362046 | 0.34066798 | 6.409436 | 0.011988 |
| PCNM 146 | 149 | 0.006463 | 0.368508 | 0.34619417 | 5.792474 | 0.01998 |
| PCNM 268 | 271 | 0.00629 | 0.374798 | 0.35156076 | 5.684306 | 0.013986 |
| PCNM 250 | 253 | 0.006253 | 0.381051 | 0.35690768 | 5.697635 | 0.014985 |
| PCNM 327 | 330 | 0.006113 | 0.387164 | 0.3621282 | 5.615927 | 0.022977 |
| PCNM 314 | 317 | 0.006039 | 0.393203 | 0.36728981 | 5.592922 | 0.015984 |
| PCNM 85 | 88 | 0.005887 | 0.39909 | 0.37231103 | 5.495743 | 0.01998 |
| PCNM 144 | 147 | 0.005851 | 0.40494 | 0.37731265 | 5.506121 | 0.021978 |
| PCNM 223 | 226 | 0.005549 | 0.41049 | 0.38201583 | 5.26189 | 0.027972 |
| PCNM 41 | 44 | 0.005539 | 0.416029 | 0.38672561 | 5.292969 | 0.021978 |
| PCNM 148 | 151 | 0.005427 | 0.421455 | 0.39133374 | 5.224546 | 0.024975 |
| PCNM 104 | 107 | 0.005337 | 0.426792 | 0.39586357 | 5.1764 | 0.014985 |
| PCNM 318 | 321 | 0.005282 | 0.432074 | 0.40035231 | 5.162009 | 0.027972 |
| PCNM 62 | 65 | 0.004777 | 0.436852 | 0.40432336 | 4.699877 | 0.024975 |
| PCNM 57 | 60 | 0.004734 | 0.441585 | 0.40826233 | 4.687767 | 0.037962 |
| PCNM 424 | 427 | 0.004252 | 0.445838 | 0.4117047 | 4.235846 | 0.044955 |
| PCNM 231 | 234 | 0.00423 | 0.450068 | 0.41513543 | 4.237953 | 0.045954 |
| PCNM 43 | 46 | 0.00422 | 0.454288 | 0.4185687 | 4.253575 | 0.03996 |
| PCNM 31 | 34 | 0.004125 | 0.458413 | 0.42191256 | 4.181389 | 0.037962 |
| PCNM 211 | 214 | 0.004072 | 0.462484 | 0.4252115 | 4.150933 | 0.030969 |
| PCNM 238 | 241 | 0.00384 | 0.466324 | 0.42827406 | 3.935471 | 0.047952 |
