# Supplementary material for: Stream fish metacommunity organisation across a Neotropical ecoregion: The role of environment, anthropogenic impact and dispersal-based processes
Source: PLoS One. 2020 May 26;15(5):e0233733. doi: 10.1371/journal.pone.0233733 (PMC7250414; doi:10.1371/journal.pone.0233733)
Supplement: S4 Table — (DOCX) [file pone.0233733.s004.docx]

**S4 Table - Spatial variables selected by forward selection procedure for alpha diversity (p <= 0.05).**

| **Variables** | **Order** | **R2** | **R2Cum** | **AdjR2Cum** | **F** | **pval** |
| --- | --- | --- | --- | --- | --- | --- |
| PCNM6 | 9 | 0.063712 | 0.063712 | 0.06211121 | 39.80755 | 0.000999 |
| PCNM1 | 4 | 0.029936 | 0.093647 | 0.09054328 | 19.28868 | 0.000999 |
| PCNM96 | 99 | 0.01962 | 0.113267 | 0.10870412 | 12.89945 | 0.001998 |
| *Betweenness centrality* | 2 | 0.017769 | 0.131036 | 0.12506357 | 11.90086 | 0.001998 |
| PCNM 4 | 7 | 0.018215 | 0.149251 | 0.14192929 | 12.43945 | 0.000999 |
| PCNM 5 | 8 | 0.015738 | 0.164989 | 0.15635063 | 10.93161 | 0.000999 |
| PCNM 58 | 61 | 0.015455 | 0.180444 | 0.17053539 | 10.91864 | 0.002997 |
| PCNM 59 | 62 | 0.012577 | 0.193021 | 0.18185138 | 9.008271 | 0.005994 |
| PCNM 44 | 47 | 0.012656 | 0.205677 | 0.19328732 | 9.193718 | 0.003996 |
| PCNM 139 | 142 | 0.011819 | 0.217496 | 0.20391053 | 8.699627 | 0.005994 |
| PCNM 85 | 88 | 0.011511 | 0.229007 | 0.21425769 | 8.585134 | 0.004995 |
| PCNM 95 | 98 | 0.011082 | 0.240089 | 0.22420261 | 8.370904 | 0.005994 |
| PCNM 232 | 235 | 0.009716 | 0.249805 | 0.23278488 | 7.420916 | 0.006993 |
| PCNM 358 | 361 | 0.009115 | 0.25892 | 0.24078126 | 7.035056 | 0.010989 |
| PCNM 123 | 126 | 0.008715 | 0.267635 | 0.24839549 | 6.794719 | 0.00999 |
| PCNM 82 | 85 | 0.008325 | 0.27596 | 0.25563586 | 6.554071 | 0.00999 |
| PCNM 155 | 158 | 0.008376 | 0.284335 | 0.26295343 | 6.659096 | 0.013986 |
| PCNM 33 | 36 | 0.007881 | 0.292217 | 0.26978692 | 6.324824 | 0.011988 |
| PCNM 62 | 65 | 0.007283 | 0.2995 | 0.27602622 | 5.895096 | 0.024975 |
| PCNM 25 | 28 | 0.007201 | 0.306701 | 0.28220276 | 5.878952 | 0.018981 |
| PCNM 441 | 444 | 0.007432 | 0.314133 | 0.28864057 | 6.122306 | 0.018981 |
| PCNM 315 | 318 | 0.007065 | 0.321198 | 0.29472033 | 5.870502 | 0.015984 |
| PCNM 16 | 19 | 0.006087 | 0.327285 | 0.29980307 | 5.094085 | 0.020979 |
| PCNM 43 | 46 | 0.005726 | 0.333011 | 0.30452791 | 4.824856 | 0.026973 |
| PCNM 46 | 49 | 0.005562 | 0.338574 | 0.30909833 | 4.717722 | 0.040959 |
| PCNM 361 | 364 | 0.005536 | 0.34411 | 0.31365785 | 4.72684 | 0.027972 |
| PCNM 20 | 23 | 0.005273 | 0.349383 | 0.31795797 | 4.530678 | 0.034965 |
| PCNM 12 | 15 | 0.005835 | 0.355218 | 0.32286335 | 5.049558 | 0.030969 |
| PCNM 50 | 53 | 0.005839 | 0.361057 | 0.32779086 | 5.090323 | 0.022977 |
| PCNM 55 | 58 | 0.005474 | 0.366531 | 0.33235109 | 4.804465 | 0.024975 |
| PCNM 8 | 11 | 0.005827 | 0.372358 | 0.33730086 | 5.152826 | 0.023976 |
| PCNM 38 | 41 | 0.004772 | 0.37713 | 0.34115232 | 4.244392 | 0.032967 |
| PCNM 2 | 5 | 0.00456 | 0.381691 | 0.34479328 | 4.078551 | 0.036963 |
| PCNM 174 | 177 | 0.004512 | 0.386202 | 0.34839572 | 4.057303 | 0.03996 |
| PCNM 142 | 145 | 0.004452 | 0.390654 | 0.35194789 | 4.025677 | 0.038961 |
