# Supplementary material for: Stream fish metacommunity organisation across a Neotropical ecoregion: The role of environment, anthropogenic impact and dispersal-based processes
Source: PLoS One. 2020 May 26;15(5):e0233733. doi: 10.1371/journal.pone.0233733 (PMC7250414; doi:10.1371/journal.pone.0233733)
Supplement: S5 Table — (DOCX) [file pone.0233733.s005.docx]

**S5 Table - List of species with number of occurrences and orders for the upper Paraná River basin.**

| Species | Order | Alfa diversity |
| --- | --- | --- |
| *Hypostomus_*cf*_ancistroides* | Siluriformes | 307 |
| *Astyanax_lacustris* | Characiformes | 288 |
| *Rhamdia_aff_quelen* | Siluriformes | 247 |
| *Astyanax_fasciatus* | Characiformes | 235 |
| *Piabina_argentea* | Characiformes | 222 |
| *Characidium_*aff*_zebra* | Characiformes | 181 |
| *Piabarchus_stramineus* | Characiformes | 156 |
| *Aspidoras_fuscoguttatus* | Siluriformes | 144 |
| *Poecilia_reticulata* | Cyprinodontiformes | 142 |
| *Hoplias_*cf*_malabaricus* | Characiformes | 132 |
| *Imparfinis_schubarti* | Siluriformes | 125 |
| *Oligosarcus_pintoi* | Characiformes | 125 |
| *Cichlasoma_paranaense* | Cichliformes | 123 |
| *Gymnotus_*cf*_sylvius* | Gymnotiformes | 121 |
| *Cetopsorhamdia_iheringi* | Siluriformes | 116 |
| *Astyanax_bockmanni* | Characiformes | 114 |
| *Serrapinnus_notomelas* | Characiformes | 110 |
| *Knodus_*cf*_moenkhausii* | Characiformes | 105 |
| *Astyanax_*cf*_paranae* | Characiformes | 100 |
| *Corydoras_aeneus* | Siluriformes | 93 |
| *Gymnotus_*cf*_carapo_australis* | Gymnotiformes | 87 |
| *Crenicichla_britskii* | Cichliformes | 81 |
| *Phalloceros_harpagos* | Cyprinodontiformes | 76 |
| *Characidium_gomesi* | Characiformes | 67 |
| *Parodon_nasus* | Characiformes | 64 |
| *Synbranchus_*cf*_marmoratus* | Synbranchiformes | 62 |
| *Hypostomus_*cf*_nigromaculatus* | Siluriformes | 59 |
| *Geophagus_*cf*_brasiliensis* | Cichliformes | 58 |
| *Hypostomus_iheringii* | Siluriformes | 57 |
| *Apareiodon_ibitiensis* | Characiformes | 55 |
| *Hemigrammus_marginatus* | Characiformes | 42 |
| *Phenacorhamdia_tenebrosa* | Siluriformes | 42 |
| *Imparfinis_mirini* | Siluriformes | 40 |
| *Eigenmannia_*cf*_trilineata* | Gymnotiformes | 38 |
| *Pyrrhulina_australis* | Characiformes | 38 |
| *Steindachnerina_insculpta* | Characiformes | 38 |
| *Hypostomus_regani* | Siluriformes | 36 |
| *Moenkhausia_sanctaefilomenae* | Characiformes | 34 |
| *Pimelodella_gracilis* | Siluriformes | 31 |
| *Bryconamericus_turiuba* | Characiformes | 28 |
| *Characidium_fasciatum* | Characiformes | 27 |
| *Trichomycterus_*cf*_brasiliensis* | Siluriformes | 26 |
| *Cyphocharax_vanderi* | Cypriniformes | 23 |
| *Eigenmannia_virescens* | Gymnotiformes | 23 |
| *Laetacara_araguaiae* | Cichliformes | 22 |
| *Moenkhausia_forestii* | Characiformes | 22 |
| *Cyphocharax_modestus* | Cypriniformes | 21 |
| *Coptodon_rendalli* | Cichliformes | 21 |
| *Imparfinis_borodini* | Siluriformes | 21 |
| *Leporinus_microphthalmus* | Characiformes | 20 |
| *Rhinolekos_arachas* | Siluriformes | 20 |
| *Oligosarcus_planaltinae* | Characiformes | 20 |
| *Apareiodon_piracicabae* | Characiformes | 19 |
| *Otothyropsis_polyodon* | Siluriformes | 19 |
| *Callichthys_callichthys* | Siluriformes | 18 |
| *Curculionichthys_insperatus* | Siluriformes | 18 |
| *Trichomycterus_candidus* | Siluriformes | 18 |
| *Aspidoras_lakoi* | Siluriformes | 17 |
| *Hypostomus_*cf*_strigaticeps* | Siluriformes | 17 |
| *Leporinus_friderici* | Characiformes | 17 |
| *Neoplecostomus_selenae* | Siluriformes | 17 |
| *Hisonotus_francirochai* | Siluriformes | 16 |
| *Hyphessobrycon_eques* | Characiformes | 16 |
| *Gymnotus_*cf*_inaequilabiatus* | Gymnotiformes | 15 |
| *Oreochromis_niloticus* | Cichliformes | 15 |
| *Melanorivulus_apiamici* | Cyprinodontiformes | 14 |
| *Planaltina_myersi* | Characiformes | 14 |
| *Sternopygus_macrurus* | Gymnotiformes | 14 |
| *Characidium_schubarti* | Characiformes | 13 |
| *Hasemania_hanseni* | Characiformes | 13 |
| *Curculionichthys_piracanjuba* | Siluriformes | 13 |
| *Hyphessobrycon_anisitsi* | Characiformes | 13 |
| *Pimelodella_avanhandavae* | Siluriformes | 13 |
| *Serrapinnus_heterodon* | Characiformes | 13 |
| *Neoplecostomus_paranensis* | Siluriformes | 12 |
| *Phalloceros_reisi* | Cyprinodontiformes | 12 |
| *Rineloricaria_pentamaculata* | Siluriformes | 12 |
| *Cetopsis_gobioides* | Siluriformes | 11 |
| *Otothyropsis_biamnicus* | Siluriformes | 10 |
| *Rineloricaria_latirostris* | Siluriformes | 10 |
| *Cambeva_davisi* | Siluriformes | 10 |
| *Rhamdiopsis_*sp. | Siluriformes | 9 |
| *Acestrorhynchus_lacustris* | Characiformes | 8 |
| *Apareiodon_affinis* | Characiformes | 8 |
| *Corydoras_difluviatilis* | Siluriformes | 8 |
| *Crenicichla_haroldoi* | Cichliformes | 8 |
| *Satanoperca_pappaterra* | Cichliformes | 8 |
| *Bryconamericus*_cf*_coeruleus* | Characiformes | 7 |
| *Bryconamericus_*cf*_iheringii* | Characiformes | 7 |
| *Crenicichla_*sp*.* "Paraná" sensu Varella 2011 | Cichliformes | 7 |
| *Hoplerythrinus_unitaeniatus* | Characiformes | 7 |
| *Pyxiloricaria_menezesi* | Siluriformes | 7 |
| *Tatia_neivai* | Siluriformes | 7 |
| *Aphyocharax_dentatus* | Characiformes | 6 |
| *Hemigrammus_parana* | Characiformes | 6 |
| *Hypostomus_margaritifer* | Siluriformes | 6 |
| *Leporinus_paranensis* | Characiformes | 6 |
| *Leporinus_striatus* | Characiformes | 6 |
| *Oligosarcus_paranensis* | Characiformes | 6 |
| *Planaltina_britskii* | Characiformes | 6 |
| *Poecilia_vivipara* | Cyprinodontiformes | 6 |
| *Melanorivulus_pictus* | Cyprinodontiformes | 6 |
| *Steindachnerina_corumbae* | Characiformes | 6 |
| *Hoplias_intermedius* | Characiformes | 5 |
| *Moenkhausia_intermedia* | Characiformes | 5 |
| *Pimelodus_maculatus* | Siluriformes | 5 |
| *Bunocephalus_larai* | Siluriformes | 4 |
| *Corydoras_flaveolus* | Siluriformes | 4 |
| *Galeocharax_gulo* | Characiformes | 4 |
| *Hypostomus_topavae* | Siluriformes | 4 |
| *Leporinus_lacustris* | Characiformes | 4 |
| *Megaleporinus_obtusidens* | Characiformes | 4 |
| *Leporinus_octofasciatus* | Characiformes | 4 |
| *Metynnis_lippincottianus* | Characiformes | 4 |
| *Prochilodus_lineatus* | Characiformes | 4 |
| *Roeboides_descalvadensis* | Characiformes | 4 |
| *Brycon_orbignyanus* | Characiformes | 3 |
| *Characidium_*aff*_lagosantense* | Characiformes | 3 |
| *Crenicichla_jaguarensis* | Cichliformes | 3 |
| *Farlowella_hahni* | Siluriformes | 3 |
| *Hoplosternum_littorale* | Siluriformes | 3 |
| *Moenkhausia_bonita* | Characiformes | 3 |
| *Psellogrammus_kennedyi* | Characiformes | 3 |
| *Rhyacoglanis_paranensis* | Siluriformes | 3 |
| *Rineloricaria_lanceolata* | Siluriformes | 3 |
| *Steindachnerina_brevipinna* | Characiformes | 3 |
| *Cambeva_diabola* | Siluriformes | 3 |
| *Trichomycterus_pauciradiatus* | Siluriformes | 3 |
| *Astyanax_biotae* | Characiformes | 2 |
| *Cichla_kelberi* | Cichliformes | 2 |
| *Cyphocharax_gillii* | Characiformes | 2 |
| *Erythrinus_erythrinus* | Characiformes | 2 |
| *Heptapterus_multiradiatus* | Siluriformes | 2 |
| *Hypostomus_albopunctatus* | Siluriformes | 2 |
| *Hypostomus_hermanni* | Siluriformes | 2 |
| *Hypostomus_paulinus* | Siluriformes | 2 |
| *Lepthoplosternum_pectorale* | Siluriformes | 2 |
| *Loricariichthys_platymetopon* | Siluriformes | 2 |
| *Megalechis_personata* | Siluriformes | 2 |
| *Otothyropsis_marapoama* | Siluriformes | 2 |
| *Parastegophilus_paulensis* | Siluriformes | 2 |
| *Phenacorhamdia_*cf*_unifasciata* | Siluriformes | 2 |
| *Piaractus_mesopotamicus* | Characiformes | 2 |
| *Pseudopimelodus_mangurus* | Siluriformes | 2 |
| *Pseudostegophilus_paulensis* | Siluriformes | 2 |
| *Serrasalmus_maculatus* | Characiformes | 2 |
| *Trachelyopterus_galeatus* | Siluriformes | 2 |
| *Ancistrus_*sp. | Siluriformes | 1 |
| *Apareiodon_vladii* | Characiformes | 1 |
| *Aphyocharax_anisitsi* | Characiformes | 1 |
| *Apistogramma_commbrae* | Cichliformes | 1 |
| *Astyanax_schubarti* | Characiformes | 1 |
| *Australoheros_tavaresi* | Cichliformes | 1 |
| *Brachyhypopomus_gauderio* | Gymnotiformes | 1 |
| *Clarias_gariepinus* | Siluriformes | 1 |
| *Cyprinus_carpio* | Cypriniformes | 1 |
| *Gymnocorymbus_ternetzi* | Characiformes | 1 |
| *Hyphessobrycon_bifasciatus* | Characiformes | 1 |
| *Pterygoplichthys_ambrosettii* | Siluriformes | 1 |
| *Melanorivulus_punctatus* | Cyprinodontiformes | 1 |
| *Microglanis_garavelloi* | Siluriformes | 1 |
| *Misgurnus_anguillicaudatus* | Cypriniformes | 1 |
| *Myloplus_tiete* | Characiformes | 1 |
| *Odontostilbe_weitzmani* | Characiformes | 1 |
| *Ossancora_eigenmanni* | Siluriformes | 1 |
| *Pamphorichthys_hollandi* | Cyprinodontiformes | 1 |
| *Paravandellia_oxyptera* | Siluriformes | 1 |
| *Pareiorhina_carrancas* | Siluriformes | 1 |
| *Phallotorynus_pankalos* | Cyprinodontiformes | 1 |
| *Salminus_hilarii* | Characiformes | 1 |
| *Schizodon_altoparanae* | Characiformes | 1 |
| *Scoloplax_empousa* | Siluriformes | 1 |
| *Serrasalmus_marginatus* | Characiformes | 1 |
| *Sorubim_lima* | Siluriformes | 1 |
| *Sternarchus_*cf*_brasiliensis* | Gymnotiformes | 1 |
| *Triportheus_nematurus* | Characiformes | 1 |
| *Xiphophorus_helleri* | Cyprinodontiformes | 1 |
