# Supplementary material for: Stream fish metacommunity organisation across a Neotropical ecoregion: The role of environment, anthropogenic impact and dispersal-based processes
Source: PLoS One. 2020 May 26;15(5):e0233733. doi: 10.1371/journal.pone.0233733 (PMC7250414; doi:10.1371/journal.pone.0233733)
Supplement: S7 Table — X1- Explanation of anthropogenic environmental gradient, X2- Explanation of natural environmental gradient, X3- Explanation of space component A—Anthropogenic environmental gradient, B—Natural environmental gradient, C—Space, D—Shared effect between anthropogenic and natural environmental gradient, E—Shared effect between anthropogenic environmental gradient and space, F—Shared effect between natural environmental gradient and space, G—Shared effect between anthropogenic, natural environmental gradient and space, H—Residuals. Bold values indicate sets of predictors that were significant (p <0.05). (DOCX) [file pone.0233733.s007.docx]

**S7 Table. Results of variance partition for alpha diversity**. X1- Explanation of anthropogenic environmental gradient, X2- Explanation of natural environmental gradient, X3- Explanation of space component A - Anthropogenic environmental gradient, B - Natural environmental gradient, C - Space, D - Shared effect between anthropogenic and natural environmental gradient, E - Shared effect between anthropogenic environmental gradient and space, F - Shared effect between natural environmental gradient and space, G - Shared effect between anthropogenic, natural environmental gradient and space, H - Residuals. Bold values indicate sets of predictors that were significant (p <0.05).

| Partition table | Df | R.square | Adj.R.square | Testable |
| --- | --- | --- | --- | --- |
| [a+d+f+g] = X1 | 12 | 0.14106 | 0.12310 | TRUE |
| [b+d+e+g] = X2 | 14 | 0.21048 | 0.19115 | TRUE |
| [c+e+f+g] = X3 | 35 | 0.39065 | 0.35195 | TRUE |
| [a+b+d+e+f+g] = X1+X2 | 26 | 0.25636 | 0.22184 | TRUE |
| [a+c+d+e+f+g] = X1+X3 | 47 | 0.43153 | 0.38196 | TRUE |
| [b+c+d+e+f+g] = X2+X3 | 49 | 0.45964 | 0.41033 | TRUE |
| [a+b+c+d+e+f+g] = All | 61 | 0.48904 | 0.42968 | TRUE |
| Individual fractions | |  |  |  |
| [a] = X1 \| X2+X3 | 12 |  | **0.01935** | TRUE |
| [b] = X2 \| X1+X3 | 14 |  | **0.04772** | TRUE |
| [c] = X3 \| X1+X2 | 35 |  | **0.20784** | TRUE |
| [d] | 0 |  | 0.01066 | FALSE |
| [e] | 0 |  | 0.05102 | FALSE |
| [f] | 0 |  | 0.01134 | FALSE |
| [g] | 0 |  | 0.08176 | FALSE |
| [h] = Residuals |  |  | 0.57032 | FALSE |
| Controlling 1 table X | |  |  |  |
| [a+d] = X1 \| X3 | 12 |  | 0.03001 | TRUE |
| [a+f] = X1 \| X2 | 12 |  | 0.03068 | TRUE |
| [b+d] = X2 \| X3 | 14 |  | 0.05838 | TRUE |
| [b+e] = X2 \| X1 | 14 |  | 0.09873 | TRUE |
| [c+e] = X3 \| X1 | 35 |  | 0.25886 | TRUE |
| [c+f] = X3 \| X2 | 35 |  | 0.21918 | TRUE |
