# Supplementary material for: Stream fish metacommunity organisation across a Neotropical ecoregion: The role of environment, anthropogenic impact and dispersal-based processes
Source: PLoS One. 2020 May 26;15(5):e0233733. doi: 10.1371/journal.pone.0233733 (PMC7250414; doi:10.1371/journal.pone.0233733)
Supplement: S8 Table — Bold values indicate predictors that were significant (p <0.05). (DOCX) [file pone.0233733.s008.docx]

**S8 Table. Regression between the variables of each predictor set and the LCBD**. Bold values indicate predictors that were significant (p <0.05).

| **Anthropogenic environmental gradient** | **Intercept** | **Std. Error** | **t value** | **P** |
| --- | --- | --- | --- | --- |
| Biodiversity threat index | -5.42E-05 | 3.34E-05 | -1.621 | 0.1055 |
| Nitrogen loading | 1.69E-05 | 1.56E-05 | 1.081 | 0.2803 |
| Phosphorus loading | -1.15E-04 | 2.25E-05 | **-5.104** | **4.52E-07** |
| Pesticide loading | 5.14E-06 | 2.10E-05 | 0.245 | 0.8065 |
| Sediment loading | 1.16E-04 | 2.62E-05 | **4.41** | **1.24E-05** |
| Human water stress | -3.18E-05 | 1.69E-05 | -1.883 | 0.0602 |
| Cropland | -4.90E-06 | 1.94E-05 | -0.252 | 0.801 |
| Livestock density | -5.35E-06 | 1.36E-05 | -0.394 | 0.6934 |
| Populacional density | -1.13E-05 | 1.34E-05 | -0.839 | 0.4016 |
| Human footprint | -2.04E-05 | 1.42E-05 | -1.439 | 0.1506 |
| Antropic use | -5.64E-05 | 1.36E-05 | **-4.151** | **3.81E-05** |
| Urban area | 1.36E-06 | 1.42E-05 | 0.095 | 0.924 |
| **Natural environmental gradient** |  |  |  |  |
| Mean Diurnal Range | 1.70E-03 | 1.07E-05 | -0.588 | 0.557099 |
| Isothermality | -1.21E-05 | 2.06E-05 | **-3.448** | **0.000606** |
| Mean Temperature of Wettest Quarter | -1.16E-04 | 3.37E-05 | -0.088 | 0.929612 |
| Mean Temperature of Driest Quarter | -2.37E-06 | 2.68E-05 | **-4.649** | **4.15E-06** |
| Annual Precipitation | -1.22E-04 | 2.62E-05 | -0.546 | 0.585014 |
| Precipitation of Wettest Month | -1.05E-05 | 1.93E-05 | 1.87 | 0.062025 |
| Precipitation of Driest Month | 5.18E-05 | 2.77E-05 | **-4.227** | **2.75E-05** |
| Precipitation of Warmest Quarter | -1.23E-04 | 2.91E-05 | **4.05** | **5.84E-05** |
| Flow accumulation | 6.58E-05 | 1.62E-05 | 1.172 | 0.241781 |
| Shreve's Hierarchy | 1.39E-05 | 1.18E-05 | 0.705 | 0.480987 |
| Strahler's Hierarchy | 9.84E-06 | 1.40E-05 | -1.894 | 0.058716 |
| Slope | -2.58E-05 | 1.36E-05 | -1.22 | 0.222858 |
| Natural forest formations | -1.97E-05 | 1.61E-05 | **2.123** | **0.03416** |
| **Spatial variables** |  |  |  |  |
| PCNM 5 | 0.00004553 | 0.00000962 | **4.733** | **0.00000283** |
| PCNM 4 | 0.00006914 | 9.502E-06 | **7.277** | **1.2E-12** |
| PCNM 10 | -6.673E-05 | 9.719E-06 | **-6.866** | **1.81E-11** |
| PCNM 6 | -6.596E-05 | 9.733E-06 | **-6.777** | **3.2E-11** |
| PCNM 1 | -5.343E-05 | 9.703E-06 | **-5.507** | **5.65E-08** |
| PCNM 7 | -3.722E-05 | 9.826E-06 | **-3.788** | **0.000169** |
| PCNM 11 | 0.00003938 | 9.644E-06 | **4.083** | **0.0000512** |
| PCNM 59 | 0.00003114 | 0.00000962 | **3.237** | **0.001283** |
| PCNM 16 | -4.077E-05 | 9.502E-06 | **-4.291** | **0.0000211** |
| PCNM 8 | -2.343E-05 | 9.638E-06 | **-2.431** | **0.015366** |
| PCNM 33 | 0.00003536 | 9.675E-06 | **3.655** | **0.000282** |
| PCNM 60 | 0.00003082 | 9.455E-06 | **3.259** | **0.001187** |
| PCNM 155 | 0.00003281 | 9.483E-06 | **3.46** | **0.000583** |
| PCNM 17 | -3.579E-05 | 0.00000965 | **-3.708** | **0.00023** |
| PCNM 247 | 0.00002871 | 9.447E-06 | **3.039** | **0.002485** |
| PCNM 232 | 0.000027 | 9.415E-06 | **2.868** | **0.004298** |
| PCNM 85 | 0.0000247 | 9.484E-06 | **2.605** | **0.00945** |
| PCNM 96 | 0.00002539 | 9.449E-06 | **2.687** | **0.00742** |
| PCNM 58 | 0.00002563 | 9.489E-06 | **2.701** | **0.007129** |
| PCNM 327 | 0.00002378 | 9.405E-06 | **2.528** | **0.011742** |
| PCNM 250 | -2.358E-05 | 9.407E-06 | **-2.506** | **0.012494** |
| PCNM 30 | -2.415E-05 | 9.586E-06 | **-2.519** | **0.012044** |
| PCNM 144 | -2.386E-05 | 9.458E-06 | **-2.523** | **0.01192** |
| PCNM 268 | 0.00002392 | 9.404E-06 | **2.543** | **0.011256** |
| PCNM 104 | 0.00002462 | 0.00000945 | **2.605** | **0.009447** |
| PCNM 62 | 0.00002127 | 0.00000948 | **2.244** | **0.025223** |
| PCNM 146 | 0.00001993 | 9.466E-06 | **2.105** | **0.035759** |
| PCNM 57 | -1.863E-05 | 0.00000952 | -1.957 | 0.050856 |
| PCNM 223 | 0.00002403 | 9.461E-06 | **2.539** | **0.011382** |
| PCNM 211 | -1.879E-05 | 9.428E-06 | **-1.993** | **0.046782** |
| PCNM 314 | 0.00002268 | 9.493E-06 | **2.389** | **0.017213** |
| PCNM 228 | 0.00001251 | 9.464E-06 | 1.322 | 0.186613 |
| PCNM 424 | 0.00002027 | 0.00000942 | **2.151** | **0.031878** |
| PCNM 148 | -2.203E-05 | 9.437E-06 | **-2.335** | **0.019912** |
| PCNM 41 | 0.00002078 | 9.522E-06 | **2.182** | **0.029529** |
| PCNM 231 | -1.964E-05 | 9.405E-06 | **-2.089** | **0.037199** |
| PCNM 82 | 0.00001945 | 9.491E-06 | **2.05** | **0.04089** |
| PCNM 107 | 0.00001948 | 0.00000948 | **2.055** | **0.040329** |
| PCNM 24 | 0.00001776 | 9.602E-06 | 1.85 | 0.064859 |
| PCNM 238 | -1.883E-05 | 9.405E-06 | **-2.002** | **0.045758** |
| PCNM 112 | 0.0000185 | 9.436E-06 | 1.96 | 0.05047 |
| PCNM 50 | 0.0000174 | 9.516E-06 | 1.829 | 0.06798 |
| PCNM 31 | -1.854E-05 | 9.492E-06 | -1.953 | 0.051325 |
