# Supplementary material for: Stream fish metacommunity organisation across a Neotropical ecoregion: The role of environment, anthropogenic impact and dispersal-based processes
Source: PLoS One. 2020 May 26;15(5):e0233733. doi: 10.1371/journal.pone.0233733 (PMC7250414; doi:10.1371/journal.pone.0233733)
Supplement: S9 Table — Bold values indicate predictors that were significant (p <0.05). (DOCX) [file pone.0233733.s009.docx]

**S9 Table. Regression between the variables of each set of predictors and alpha diversity**. Bold values indicate predictors that were significant (p <0.05).

| **Anthropogenic environmental gradient** | **Intercept** | **Std. Error** | **t value** | **P** |
| --- | --- | --- | --- | --- |
| Biodiversity threat index | 7.31E-01 | 6.11E-01 | 1.197 | 0.2319 |
| Nitrogen loading | -3.42E-01 | 2.85E-01 | -1.2 | 0.2308 |
| Phosphorus loading | 1.62E+00 | 4.10E-01 | **3.961** | **8.41E-05** |
| Pesticide loading | -5.06E-01 | 3.83E-01 | -1.32 | 0.1872 |
| Sediment loading | -5.03E-01 | 4.79E-01 | -1.049 | 2.95E-01 |
| Human water stress | 2.55E-01 | 3.09E-01 | 0.826 | 0.4093 |
| Cropland | -1.93E-01 | 3.55E-01 | -0.544 | 0.5866 |
| Livestock density | -4.71E-01 | 2.48E-01 | -1.9 | 0.0579 |
| Populacional density | 3.87E-01 | 2.45E-01 | 1.576 | 0.1156 |
| Human footprint | 3.25E-01 | 2.59E-01 | 1.255 | 0.2101 |
| Antropic use | 1.29E-01 | 2.48E-01 | **0.518** | 6.05E-01 |
| Urban area | -5.41E-01 | 2.60E-01 | **-2.085** | **0.0376** |
| **Natural environmental gradient** |  |  |  |  |
| Mean Diurnal Range | -1.184961 | 0.376577 | **-3.147** | **0.001737** |
| Isothermality | 2.761828 | 0.617082 | **4.476** | **9.20E-06** |
| Mean Temperature of Wettest Quarter | 0.805057 | 0.489629 | 1.644 | 0.100681 |
| Mean Temperature of Driest Quarter | 1.902427 | 0.479627 | **3.966** | **8.22E-05** |
| Annual Precipitation | -0.045721 | 0.352259 | -0.13 | 0.896775 |
| Precipitation of Wettest Month | -0.983546 | 0.507312 | -1.939 | 0.053024 |
| Precipitation of Driest Month | 2.104863 | 0.532565 | **3.952** | **8.71E-05** |
| Precipitation of Warmest Quarter | -0.517614 | 0.297106 | -1.742 | 0.082014 |
| Flow accumulation | 0.464865 | 0.216337 | **2.149** | **0.032069** |
| Shreve's Hierarchy | -0.314247 | 0.255257 | -1.231 | 0.218792 |
| Strahler's Hierarchy | 0.857311 | 0.248963 | **3.444** | **0.000616** |
| Slope | 0.457505 | 0.295364 | 1.549 | 0.121945 |
| Natural forest formations | -0.002561 | 0.264956 | -0.01 | 0.992292 |
| Natural non-forest formations | -0.35635 | 0.211713 | -1.683 | 0.092886 |
| **Spatial variables** |  |  |  |  |
| PCNM6 | 1.2941 | 0.179 | **7.231** | **1.61E-12** |
| PCNM1 | 0.9905 | 0.1825 | **5.427** | **8.59E-08** |
| PCNM96 | -0.6602 | 0.1774 | **-3.722** | **0.000218** |
| *Betweenness centrality* | 0.9011 | 0.1868 | **4.824** | **0.00000182** |
| PCNM 4 | -0.8335 | 0.1778 | **-4.688** | **0.00000348** |
| PCNM 5 | -0.7469 | 0.179 | **-4.172** | **0.0000351** |
| PCNM 58 | -0.6754 | 0.1776 | **-3.802** | **0.000159** |
| PCNM 59 | -0.6147 | 0.1795 | **-3.425** | **0.00066** |
| PCNM 44 | -0.5514 | 0.1783 | **-3.093** | **0.00208** |
| PCNM 139 | -0.5695 | 0.1775 | **-3.209** | **0.00141** |
| PCNM 85 | -0.5624 | 0.1773 | **-3.172** | **0.0016** |
| PCNM 95 | 0.4461 | 0.1781 | **2.505** | **0.012548** |
| PCNM 232 | -0.5193 | 0.1761 | **-2.949** | **0.003325** |
| PCNM 358 | -0.4803 | 0.1784 | **-2.692** | **0.007323** |
| PCNM 123 | 0.4306 | 0.1771 | **2.432** | **0.015331** |
| PCNM 82 | -0.3669 | 0.1777 | **-2.064** | **0.03947** |
| PCNM 155 | -0.5017 | 0.1799 | **-2.788** | **0.005483** |
| PCNM 33 | -0.4726 | 0.1783 | **-2.651** | **0.008266** |
| PCNM 62 | -0.4864 | 0.1771 | **-2.747** | **0.006214** |
| PCNM 25 | 0.4748 | 0.1779 | **2.669** | **0.007835** |
| PCNM 441 | 0.4173 | 0.1798 | **2.32** | **0.020682** |
| PCNM 315 | -0.4488 | 0.1761 | **-2.548** | **0.011107** |
| PCNM 16 | 0.4513 | 0.1789 | **2.522** | **0.011941** |
| PCNM 43 | -0.4169 | 0.1793 | **-2.325** | **0.020438** |
| PCNM 46 | 0.4753 | 0.1787 | **2.659** | **0.008056** |
| PCNM 361 | 0.3877 | 0.1764 | **2.198** | **0.028389** |
| PCNM 20 | -0.4191 | 0.1791 | **-2.34** | **0.019626** |
| PCNM 12 | 0.4853 | 0.1794 | **2.705** | **0.00704** |
| PCNM 50 | -0.4378 | 0.179 | **-2.446** | **0.014766** |
| PCNM 55 | -0.4091 | 0.1794 | **-2.28** | **0.022959** |
| PCNM 8 | 0.446 | 0.1796 | **2.483** | **0.013322** |
| PCNM 38 | 0.3932 | 0.1788 | **2.199** | **0.028292** |
| PCNM 2 | 0.3717 | 0.1789 | **2.077** | **0.038262** |
| PCNM 174 | -0.3728 | 0.1783 | **-2.091** | **0.03699** |
| PCNM 142 | -0.3627 | 0.1807 | **-2.006** | **0.045301** |
